# Exploring the epigenetic role of the circular RNA ciR-01114 in porcine ovarian cell functions

**DOI:** 10.1007/s11033-025-11126-6

**Published:** 2025-10-11

**Authors:** Zuzana Fabová, Barbora Loncová, Abdel Halim Harrath, Anouar Feriani, Alexander V. Sirotkin

**Affiliations:** 1 Department of Zoology, College of Science, King Saud University, Riyadh, 11451 Saudi Arabia; 2 Laboratory of Biotechnology and Biomonitoring of the Environment and Oasis Ecosystems, University of Gafsa, Gafsa, 2112 Tunisia; 3 Department of Zoology and Anthropology, Constantine the Philosopher University, Tr. A. Hlinku 1, 949 74, Nitra, Slovakia

**Keywords:** CircRNA, Ovary, Apoptosis, Proliferation, Steroidogenesis

## Abstract

**Background:**

Circular RNAs (circRNAs) are emerging epigenetic regulators in reproductive biology, but specific functions of many ovarian circRNAs remain uncharacterized. This study investigates the functional significance of circRNA ciR-01114, previously identified in porcine ovarian tissue, in modulating ovarian cell functions.

**Methods and results:**

To evaluate the impact of ciR-01114 on the regulation of basic porcine ovarian granulosa cell functions, we performed experiments to increase (using an overexpressing vector) and decrease (using a shRNA vector) the expression of this circRNA. We assessed the relative expression levels of ciR-01114, cell viability, proliferation (measured by the accumulation of PCNA and cyclin B1), cytoplasmic (assessed via the accumulation of bax and caspase-3), and nuclear (DNA fragmentation) apoptosis, and the release of progesterone, testosterone, estradiol, IGF-I, and oxytocin. The overexpression of ciR-01114 significantly enhanced cell viability, proliferation, and the release of progesterone, testosterone, estradiol, and oxytocin, while suppressing apoptosis. Conversely, ciR-01114 knockdown yielded opposing effects.

**Conclusions:**

These preliminary findings suggest that ciR-01114 may regulate porcine ovarian cell functions, including cell cycle progression, apoptosis, and secretory activity. However, further validation and in-depth studies are necessary to confirm these observations and fully understand the mechanisms underlying ciR-01114’s role in ovarian biology.

## Introduction

Over the past decade, the extensive study and application of epigenetic regulators in reproductive processes has led to rapid advancements in reproductive biology, assisted reproduction, and animal production. Among these regulators, circular RNAs (circRNAs) have emerged as key modulators of gene expression in reproductive processes. CircRNAs are a class of noncoding RNAs characterized by their covalently closed-loop structures, which differentiate them from linear RNAs [1]. This unique structure makes circRNAs resistant to exonuclease degradation, contributing to their stability and potential regulatory roles in various biological processes such as controlling gene splicing and transcription, serving as miRNA and protein sponges, interacting with proteins, and acting as protein-coding circRNAs [2, 3].

CircRNAs have been implicated in normal and pathological ovarian functions across multiple species, including humans, mice, cattle, and pigs [4]. Their dysregulation has been associated with ovarian diseases such as ovarian cancer, polycystic ovary syndrome (PCOS), and primary ovarian insufficiency (POI), suggesting their potential as diagnostic biomarkers and therapeutic targets [4–7]. Recent studies have demonstrated that circRNAs act as crucial mediators of signal transduction pathways by regulating signaling molecules and downstream targets. They can modulate the Wnt/β-catenin, PI3K/AKT, and ERK1/2 pathways, influencing ovarian cellular responses such as growth, differentiation, and apoptosis [5, 8]. For instance, circRHOBTB3 has been shown to suppress cell proliferation, metastasis, and glycolysis via inactivating the PI3K/AKT pathway in ovarian cancer [9]. Similarly, circFBXO7 functions as a tumor suppressor by modulating Wnt/β-catenin signaling and affecting cellular proliferation, migration, and invasion, homeostasis, also primarily studied in human ovarian cancer cells [10]. Additionally, circRNAs play a pivotal role in oocyte maturation by regulating meiotic progression. Moreover, circRNAs interact with miRNAs and RNA-binding proteins to modulate the expression of key maturation-related genes such as BMP15 and GDF9, which are critical for oocyte competence [11]. CircRNA-mediated regulation of mitochondrial activity and oxidative stress further contributes to oocyte quality and developmental potential, emphasizing their role in female fertility [4, 12]. In ovarian follicle development, circRNAs regulate granulosa cell function and follicular selection through miRNA sponging and modulation of gene expression [13]. For example, cicrCFAP20DC and cicrWRNIP1 have been shown to influence follicle development by enhancing granulosa cell proliferation in goats [14] and chickens [15]. Moreover, circSLC41A1, circRALGPS2, and aplacirc_13267 regulate apoptosis-related pathways, influencing follicular atresia and survival in pigs [16], chickens [17], and ducks [18]. Additionally, circRNAs have been found to influence the expression of FSHR and steroidogenesis-related genes such as CYP19A1 and CYP11A1, affecting hormone secretion and follicle maturation [19, 20]. A study showed that circRPS19 enhances chicken granulosa cell proliferation and steroid hormone synthesis by sponging miR-218-5p as a ceRNA, thus upregulating INHBB expression [21]. This finding highlights the role of circRNAs in modulating granulosa cell functions through miRNA-mediated pathways, which may have parallels in other species, including pigs. Finally, in our previous study, we demonstrated that ciR-00596 and ciR-00646 may impact ovarian granulosa cell proliferation, apoptosis, and secretory activity of porcine ovarian cells [22].

The expression of ciR-01114 in the porcine ovary has also been reported [23], but its specific role in ovarian function remains unclear. To address this knowledge gap, the general aim of the present study was to examine the role of ciR-01114 in regulating the fundamental functions of ovarian cells. For this purpose, we cultured porcine ovarian granulosa cells and examined the effects of ciR-01114 overexpression and knockdown on cell viability, proliferation, apoptosis, and the release of key reproductive hormones, including progesterone, testosterone, estradiol, IGF-I, and oxytocin. These hormones are critical regulators of ovarian folliculogenesis, follicular survival, ovulation, and reproductive lifespan [24]. Understanding the contribution of ciR-01114 to ovarian function could provide valuable insights into its potential role in reproductive health and disease.

## Materials and methods

### Knockdown and overexpression plasmid construction

Short hairpin RNA (shRNA) targeting the back-splice junction (BSJ) region of ciR-01114 (designated sh-ciR-01114) was cloned and inserted into the pGPU6/green fluorescent protein (GFP)/Neo vector (GenePharma, Shanghai, China). A negative control shRNA (sh-NC) was also constructed using the same vector but contained sequences that did not complement the BSJ region. The full-length sequence of ciR-01114 was inserted into the pEX-3 (pGCMV/MCS/Neo) vector to generate the overexpression vector. An empty pEX-3 vector served as a negative control. The sequences for all the constructs (refer to Table 1) were obtained from GenePharma. The control groups included cells that were either not transfected or were transfected with negative controls (NCs). The knockdown and overexpression efficiencies of ciR-01114 were assessed using reverse transcription-quantitative polymerase chain reaction (RT‒qPCR).

**Table 1:** Nucleotide sequences for ShRNA expression vectors and the overexpression vector for ssc-ciR-01114

Construct type	Sequence name	Strand	Sequence (5′–3′)
shRNA expression vector	sh-ssc-ciR-01114	Sense	CACCGCAAAGCCTGGCCTGGGAACCTTCAAGAGAGGTTCCCAGGCCAGGCTTTGCTTTTTTG
		Antisense	GATCCAAAAAAGCCAAAGCCTGGCCTGGGAACCTCTCTTGAAGGTTCCCAGGCCAGGCTTTGC
	shRNA negative control	Sense	CACCGTTCTCCGAACGTGTCACGTTTCAAGAGAACGTGACACGTTCGGAGAATTTTTTG
		Antisense	GATCCAAAAAATTCTCCGAACGTGTCACGTTCTCTTGAAACGTGACACGTTCGGAGAAC
Overexpression vector	ssc-ciR-01114	-	CCTGGCCTGGGAACCTCCATATGCCTCGGGTATGGCCCTAAAAGTAAAAAGGAGCAAGGATTCTAGCGG AGAGAAAACACAGGGTGGGGAGCAAAG

### Rationale for Porcine granulosa cell model

Porcine ovarian granulosa cells were selected as a model system for this study due to their significant physiological and functional similarities to human ovarian granulosa cells, making them a valuable tool for translational reproductive research. Pigs and humans share comparable ovarian structures and endocrine functions, including similar ovarian follicular development and steroid hormone production mechanisms [25]. Studies have demonstrated that porcine granulosa cells exhibit gene expression profiles and steroidogenic pathways akin to those in humans, facilitating the extrapolation of findings to human applications. Therefore, insights gained from porcine granulosa cells can enhance our understanding of human ovarian physiology and potential therapeutic approaches [26]. The ovarian structure and follicular development in pigs closely resemble those of humans, with both species exhibiting similar endocrine regulatory mechanisms, including steroid hormone production and folliculogenesis. The porcine ovarian follicular environment, hormonal regulation, and granulosa cell function share significant similarities with human reproductive physiology, making them a suitable model for studying ovarian folliculogenesis, steroidogenesis, and oocyte maturation. Moreover, key genes and regulatory pathways involved in follicular growth, steroidogenesis, and apoptosis in porcine granulosa cells have been shown to exhibit high homology with those in human granulosa cells, allowing findings from porcine models to be extrapolated to human reproductive biology [27]. This high degree of genetic and molecular conservation enhances the translational potential of findings from porcine models to human reproductive biology and related disorders, such as polycystic ovary syndrome (PCOS) and ovarian insufficiency, which often involve dysregulation of granulosa cell function. While human granulosa cells are typically obtained from follicular aspirates during in vitro fertilization (IVF), porcine granulosa cells offer a more readily available and abundant cell source from slaughterhouse-derived ovaries, eliminating ethical concerns associated with human tissue collection and allowing for a more consistent experimental setup. By investigating ciR-01114 in porcine granulosa cells, we aim to gain insights into its potential regulatory role in human ovarian physiology, particularly given that circRNAs have been implicated in ovarian pathophysiology across species. The controlled in vitro environment afforded by the porcine granulosa cell model allows for systematic analysis of the impact of ciR-01114 on cellular proliferation, apoptosis, and steroid hormone secretion. The results of this study, while conducted in a porcine model, contribute to a broader understanding of ovarian physiology and may serve as a basis for future studies in human granulosa cells or inform potential therapeutic strategies for human reproductive conditions.

### Preparation, transfection, culture, and processing of ovarian granulosa cells

Twenty ovaries from prepubertal Landrace gilts (aged 6–8 months) were obtained from the Chovmat F.U. slaughterhouse in Rastislavice, Slovakia. These ovaries were maintained in thermoses containing a saline solution (0.9% NaCl) at room temperature and processed within six hours post-slaughter. Porcine ovarian granulosa cells were isolated from ovarian follicles (measuring 4.5–6.5 mm in diameter) that exhibited no signs of atresia such as weak vascularization, a thin follicular wall, and pale follicular fluid. The cells were aspirated using a syringe and subsequently isolated *via* centrifugation at 1,500 rpm for 10 min. Following isolation, the granulosa cells were washed in sterile DMEM/F12 1:1 medium (BioWhittakerTM; Lonza, Verviers, Belgium) and resuspended in the same medium supplemented with 10% fetal calf serum (BioWhittakerTM) and 1% antibiotic-antimycotic solution (Sigma‒Aldrich, St. Louis, MO, USA). The cell count was determined using an automated cell counter (Thermo Fisher Scientific, Inc., Waltham, MA, USA) and adjusted to a concentration of 1 × 10^6^ cells/ml. The cell suspension was distributed into culture plates for subsequent assays: 24-well plates (NuncTM, Roskilde, Denmark; 1 ml/well) for ELISA, 96-well plates (Brand^®^, Wertheim, Germany; 100 µl/well) for terminal deoxynucleotidyl transferase dUTP nick-end labeling (TUNEL) assay, and 16-well chamber slides (Nunc Inc., International, Naperville, IL, USA; 200 µl/well) for immunocytochemistry. The cells were cultured at 37.5 °C in an atmosphere of 5% CO2 until they reached 75% confluence, typically within 2–3 days.

Following preculture, the cells were transfected with a knockdown plasmid (sh-ciR-01114) and overexpression plasmid (ciR-01114) along with their respective negative controls (NCs). According to the manufacturer’s instructions, the transfections were performed using Lipofectamine^®^ RNAiMAX transfection reagent (Invitrogen, Carlsbad, CA, USA), with a final plasmid concentration of 40 nM for each transfection. The control groups included nontransfected cells and cells transfected with negative control plasmids. Posttransfection, the cells were cultured for an additional 48 h to allow for plasmid expression. Subsequently, the medium from the 24-well plates was collected and stored at − 20 °C for further ELISA analysis. Cells from the 16-well chamber slides and 96-well plates were immediately processed for immunocytochemistry, Trypan blue exclusion tests, TUNEL assays, and RT‒qPCR.

### Cell viability test

Cell viability was assessed using a Trypan blue (0.4%) exclusion test as previously described [28, 29]. Briefly, the medium was removed from the culture plates following the incubation of granulosa cells. The cell monolayer was then subjected to Trypan blue staining (Sigma‒Aldrich) for 15 min. After staining, the cells were fixed with 4% paraformaldehyde for 30 min. After fixation, the plates were washed with saline solution and examined under a microscope at 400× magnification. The proportion of dead (stained) cells to the total number of cells was subsequently calculated.

### TUNEL assay

DNA fragmentation in cell culture was assessed using the TUNEL assay (HT TiterTACS™ Apoptosis Detection Kit; Trevigen, Gaithersburg, MD, USA) following the manufacturer’s guidelines. The absorbance of TUNEL-positive cells was measured at 450 nm using an ELISA reader (Thermo Fisher Scientific, Inc.) after the addition of 0.2 N HCl. For the negative control, cells were labeled without terminal deoxynucleotidyl transferase (TdT). Positive controls were generated by treating cells with TACS-Nuclease for one hour at 37 °C before exposure to hydrogen peroxide. The proportion of DNA-fragmented cells to the total cell count was then calculated.

### Immunocytochemical analysis of proliferation and apoptosis markers

Immunocytochemistry was used to detect the presence of proliferation-related proteins (PCNA and cyclin B1) and apoptosis-related proteins (bax and caspase-3) as previously described [30]. Primary mouse monoclonal antibodies against PCNA, cyclin B1, bax, or caspase-3 (diluted 1:500 in PBS; Santa Cruz Biotechnology, Inc., CA, USA) were used. Secondary antibodies used included horseradish peroxidase (HPR)-coupled anti-mouse IgG (diluted 1:1,000; Santa Cruz Biotechnology, Inc.) and anti-mouse IgG (Sigma‒Aldrich) labeled with CruzFluor™ 594 (CFL 594, diluted 1:500). The HPR-labeled cells were stained with 3,3’-diaminobenzidine (DAB) substrate (Roche Diagnostics GmbH, Mannheim, Germany). For cells labeled with CFL 594, VECTASHIELD Antifade Mounting Medium with 4′,6-diamidino-2-phenylindole (DAPI) (Vector Laboratories, Inc., Burlingame, CA, USA), a selective stain for cell nuclear DNA, was used. Fluorescence microscopy detected the DAPI- and CFL 594-labeled secondary antibodies. To ensure the specificity and validity of the immunocytochemistry results, several important controls were implemented. Negative controls, consisting of cells incubated with secondary antibody only, were included to assess for any non-specific secondary antibody binding. This helps to rule out false-positive signals. Additionally, to control for potential artifacts arising from the DAB staining procedure, a control using cells treated with DAB substrate alone (without primary or secondary antibodies) was also included. This helps to verify that the DAB staining itself isn’t producing any background signal. The brown coloration of DAB determined the proportion of stained cells and the localization of intracellular molecules in the presence of peroxidase or the red fluorescence emitted by the CFL 594 label. Observations were made using light or fluorescence microscopy (Leica Microsystems, Wetzlar, Germany) and IM500 Leica software. The proportion of stained cells to the total cell count was calculated.

### RT‒qPCR

Total RNA from the transfected granulosa cells was extracted using the TRIzol reagent (Invitrogen) according to the manufacturer’s instructions. The quantity and quality of the extracted RNA were assessed using a UV spectrophotometer (Bio-Rad, Inc., Hercules, CA, USA). The RNA was reverse transcribed into cDNA using the PrimeScript RT reagent kit (Takara Bio, Inc., Shiga, Japan) following the manufacturer’s protocol. qPCR was performed using the SYBR Premix Ex Taq II kit (TaKaRa, Dalian, China) on an ABI 7500 Fast apparatus (Thermo Fisher Scientific, Inc.). The qPCR amplification protocol included initial denaturation and activation of Taq DNA polymerase at 95 °C for 7 min, followed by 40 cycles of denaturation at 95 °C for 10 s, annealing at 60 °C for 10 s, and extension at 72 °C for 10 s. GAPDH was used as an internal control, and relative gene expression was calculated using the 2^–ΔΔCt^ method [31]. The primer sequences (Table 2) were designed and synthesized by GenePharma. Each sample was analyzed in triplicate from the same RNA preparation and average values were determined. While qPCR amplification efficiency was confirmed to be within the acceptable range of 95–105% using serially diluted cDNA, the specific data required for precise primer efficiency calculation were not retained during the experimental process. This confirmation of acceptable amplification efficiency was performed internally to ensure the reliability of our qPCR results.

**Table 2:** The sequences of gene primers for RT-qPCR

Gene symbol	Primer sequence	
ssc-ciR-01114	Forward	5′-GGAGCAAGGATTCTAGC-3′
	Reverse	5′-CCATACCCGAGGCATAT-3′
GAPDH	Forward	5′-GAAGGTGAAGGTCGGAGT-3′
	Reverse	5′-AAGATGGTGATGGGATTTC-3′

### Enzyme-linked immunosorbent assay (ELISA)

The concentrations of progesterone, testosterone, 17β-estradiol, IGF-I, and oxytocin were measured in 25- or 100-µl aliquots of the incubation medium using enzyme-linked immunosorbent assays (ELISAs) according to the manufacturer’s instructions. The ELISA kits for progesterone, testosterone, 17β-estradiol, and IGF-I were obtained from LDN Immunoassays and Services (Nodhorn, Germany), while the oxytocin ELISA kit was sourced from Abcam (Cambridge, UK). The characteristics of these assays are detailed in Table 3. The accuracy of the ELISAs was validated for culture medium samples through dilution experiments. To ensure the accuracy and reliability of the ELISA results, several crucial controls were implemented. A blank control, consisting of wells without sample or antibody, was included to determine the background signal and account for any absorbance from the well itself or the reagents. Negative controls, using samples known to lack the target analyte, were run to identify any non-specific binding and ensure that the assay wasn’t producing false-positive readings. Positive controls, consisting of samples with known concentrations of the target analyte, were included to verify assay sensitivity and accuracy and to confirm that the assay was working as expected. A calibration curve using known concentrations of the analyte was constructed for each assay to ensure linearity and reproducibility across the measured range. The accuracy of the ELISAs was further validated for culture medium samples through dilution experiments, assessing the parallelism of the assay response in the diluted samples compared to the standard curve.

**Table 3:** Characteristics of the immunoassays used in experiments

Substance assayed	Specificity of assay (cross-reactivity of antiserum)	Sensitivity of assay (ng/ml)	Coefficient of variation (%)	
			Intra-assay	Inter-assay
**Progesterone**	≤ 1.1% with 11-desoxycorticosterone, ≤ 0.35% with pregnenolone, ≤ 0.30% 17α-OH with progesterone, ≤ 0.20% with corticosterone, ˂0.10% with estriol, 17β-estradiol, testosterone, cortisone and 11-desoxycortisol, ˂0.02% with DHEA-S and cortisol	0.045	5.4	5.59
**Testosterone**	≤ 3.3% with 11β-hydroxytestosterone and 19-nortestosterone, ≤ 0.9% with androstenedione, ≤ 0.8% with 5α-dihydrotestosterone, and ˂0.1% with 17α-methyltestosterone, epitestosterone, estradiol, progesterone, cortisol, estrone, and danazol	0.083	4.16	4.73
***17β*** **-estradiol**	≤ 9.5% with fulvestrant, ≤ 4.2% with estrone, ≤ 3.8% with E2-3-glucuronide, ≤ 3.6% with E2-3-sulphate, ≤ 0.4% with estriol, ˂0.1% with androstenedione, 17-hydroxyprogesterone, corticosterone, pregnenolone, E2-17-glucuronide, progesterone, and testosterone	0.0062	6.4	4.5
**IGF-I**	100% with IGF-I, ≤ 3.3% with insulin, and 1.02% with IGF-II	9.75	7.39	12.63
**Oxytocin**	100% with oxytocin, 7.5% with arg8-vasotocin, 7.0% with mesotocin, and ˂0.2% with ser4,lle8-oxytocin, TRH, somatostatin, met-enkephalin, VIP, lys8-vasopressin and arg8-vasopressin	0.015	12.6	11.8

### Statistical analysis

The data from this study are presented as the average values obtained from three independent experiments conducted on different days, each using distinct sets of granulosa cells isolated from a minimum of six ovaries. Each experimental group comprised four culture wells containing ovarian granulosa cells. For the Trypan blue exclusion test, viability rates were calculated for a minimum of 100 cells per well. Immunocytochemical analysis was used to determine the percentage of antigen-positive cells from a minimum of 1,000 cells per well. In the ELISAs, nonspecific background values (less than 10% of the total values) were subtracted from the corresponding values measured for media-containing cells. The secretion rates of the substances were calculated per 1 × 10^6^ viable cells per day. Statistical analysis was performed using the Shapiro-Wilk normality test, Student’s t test, and one-way ANOVA with Tukey’s post hoc test, employing SigmaPlot 11.0 (Systat Software, GmbH, Erkrath, Germany). Differences were considered statistically significant at a P value of less than 0.05 (*P* < 0.05).

## Results

### Evaluation of transfection efficiency

**Fig. 1:**
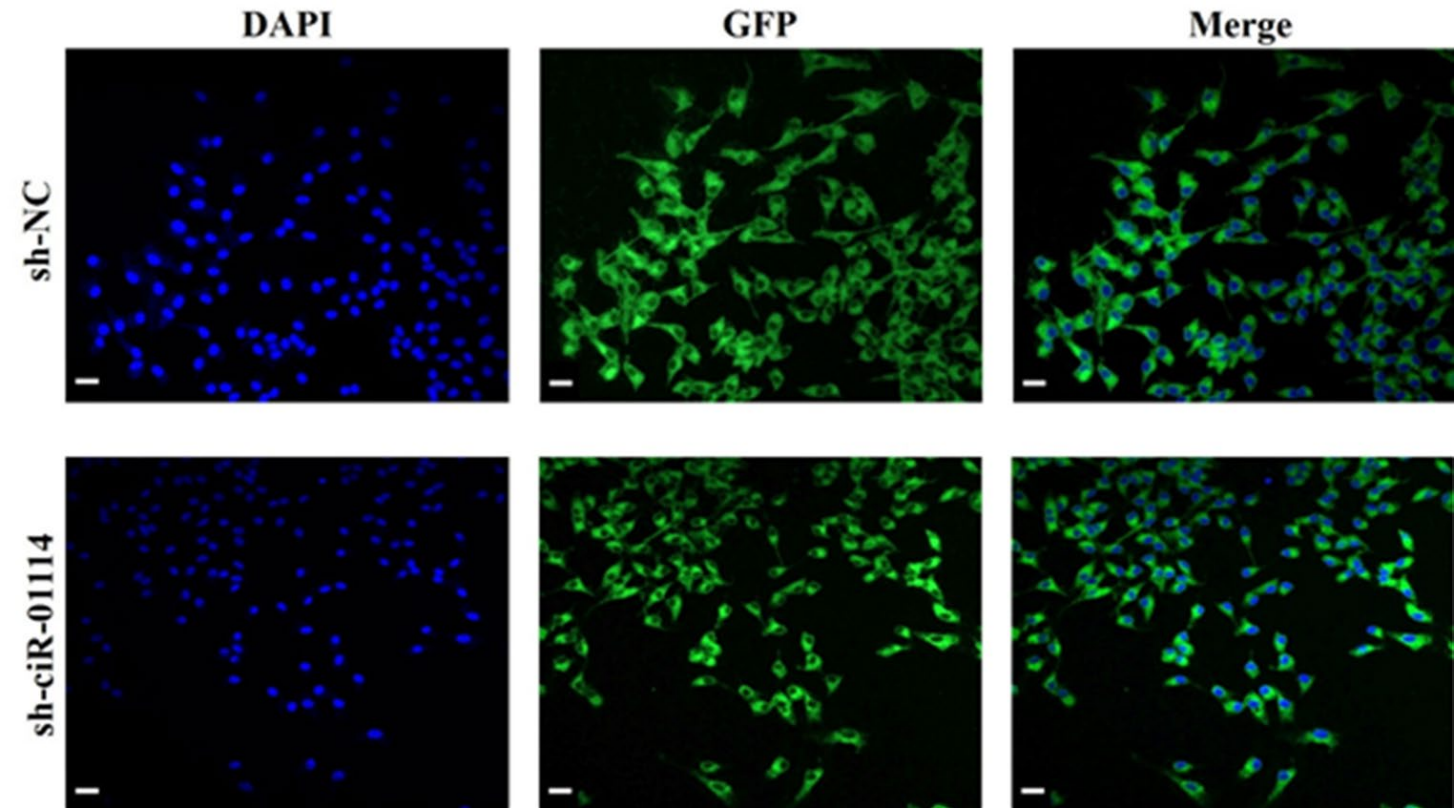
Transfection efficiency evaluated according to the expression of green fluorescent protein (GFP) in porcine granulosa cells transfected with pGPU6/GFP/Neo-sh-ciR-01114 and pGPU6/GFP/Neo-sh-negative control (NC). Fluorescence microscopy analysis of GFP-positive cells. DAPI was used to stain the cellular nuclei. Scale bars: 1 cm = 20 μm

**Fig. 2:**
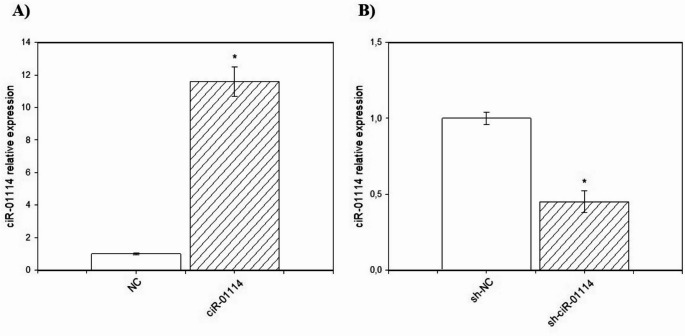
Evaluation of ciR-01114 expression levels using reverse transcription-quantitative polymerase chain reaction (RT‒qPCR) in cells transfected with the ciR-01114 overexpression vector [**A**], ciR-01114 knockdown (shRNA) vector [**B**], or the corresponding negative controls (NCs) [**A**, **B**]. The values are means ± SEMs. Significance vs. control: *, *P* < 0.05

In this study, porcine ovarian granulosa cells were transfected with a ciR-01114-overexpressing vector and shRNA knockdown vector for ciR-01114 (sh-ciR-01114), as well as their respective negative controls (NCs). Transfection efficiency was evaluated using two methods. (1) More than 86% of the cells contained GFP inserted into the pGPU6/GFP/Neo-sh-NC plasmid construct (Fig. 1). (2) RT‒qPCR was used to verify that ciR-01114 integration and expression levels increased in granulosa cells after ciR-01114 overexpression but decreased after transfection with sh-ciR-01114 (Fig. 2A, B).

*Effects of ciR-01114 overexpression and knockdown on the viability*,* proliferation*,* and apoptosis of porcine ovarian granulosa cells*.

**Fig. 3:**
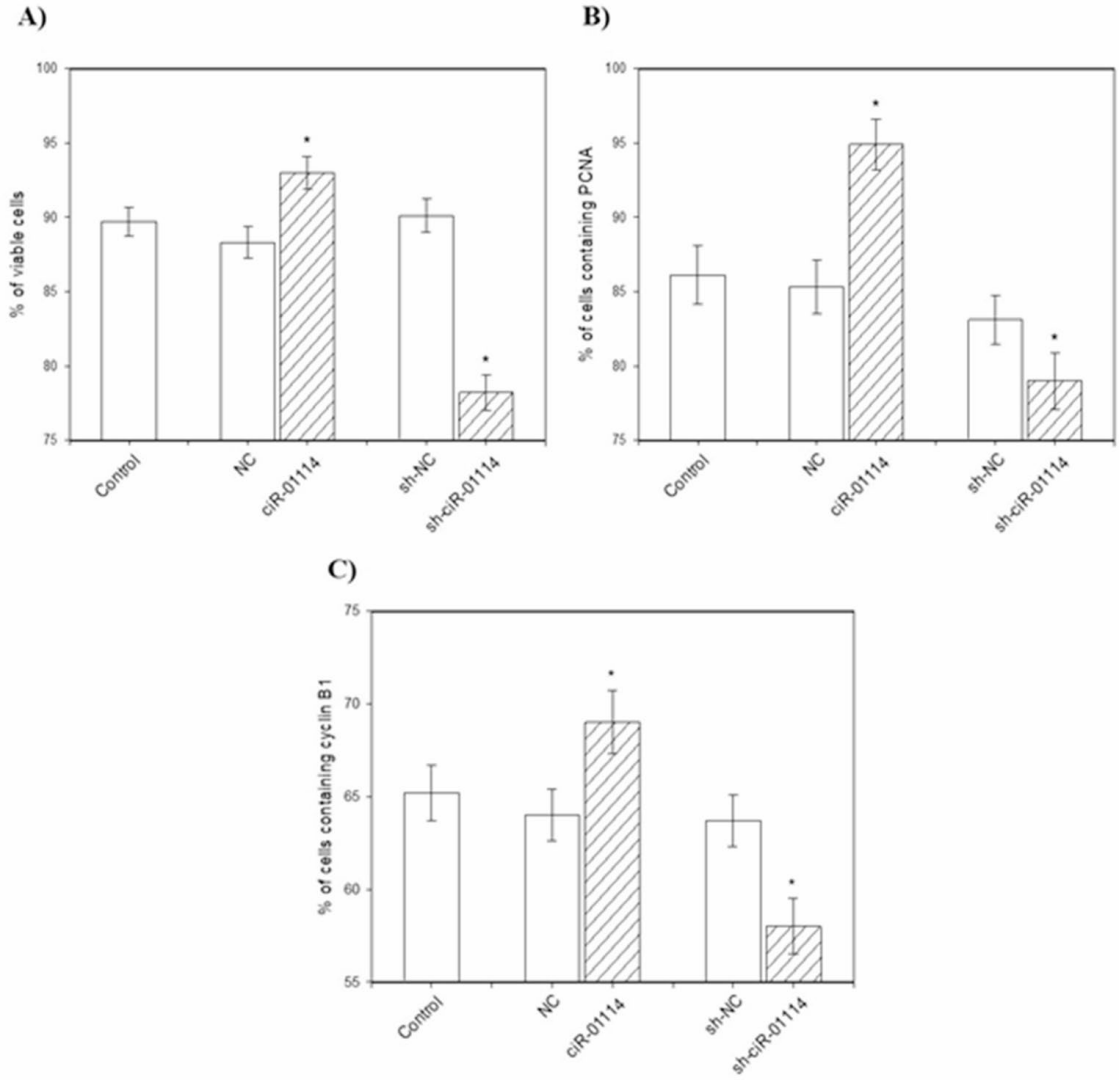
Effects of ciR-01114 overexpression and ciR-01114 suppression on cell viability [**A**] and proliferation (accumulation of PCNA [**B**] and cyclin B1 [**C**]) in cultured porcine ovarian granulosa cells. The results show (*) the effects of ciR-01114 overexpression or ciR-01114 suppression, with a significant (*P* < 0.05) difference between cells transfected with an empty pEX-3 vector as a negative control (NC) or the shRNA negative control (sh-NC) and cells transfected with the ciR-01114 overexpression vector or ciR-01114 shRNA vector. The data are expressed as means ± SEMs from at least 3 independent experiments

**Fig. 4:**
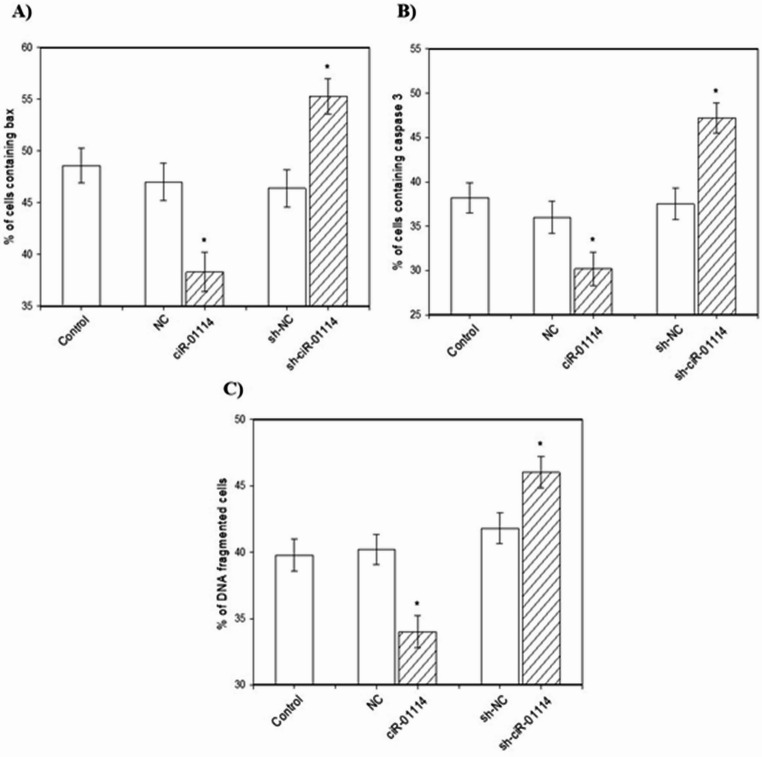
Effects of ciR-01114 overexpression and ciR-01114 suppression on cell apoptosis (accumulation of bax [**A**], caspase-3 [**B**], and TUNEL-positive cells [**C**]) in cultured porcine ovarian granulosa cells. The results show (*) the effects of ciR-01114 overexpression or ciR-01114 suppression, with a significant (*P* < 0.05) difference between cells transfected with an empty pEX-3 vector as a negative control (NC) or the shRNA negative control (sh-NC) and cells transfected with the ciR-01114 overexpression vector or ciR-01114 shRNA vector. The results are expressed as means ± SEM from at least 3 independent experiments

Transfection of cultured porcine granulosa cells with the ciR-01114 overexpression vector increased cell viability and the amount of proliferation-related proteins (PCNA and cyclin B) (Figs. 3A, B, C). Furthermore, ciR-01114 overexpression reduced the amount of apoptosis-related proteins (bax and caspase-3) and the accumulation of cells with fragmented DNA (TUNEL-positive cells) (Figs. 4A, B, C). However, sh-ciR-01114 decreased cell viability and the levels of the proliferation-related proteins PCNA and cyclin B1 (Figs. 3A, B, C). Sh-ciR-01114 increased the levels of the proapoptotic proteins bax and caspase-3 (Figs. 4A, B) and the percentage of TUNEL-positive cells (Fig. 4C).

### Effects of ciR-01114 overexpression and knockdown on the secretory activity of Porcine ovarian granulosa cells

**Fig. 5:**
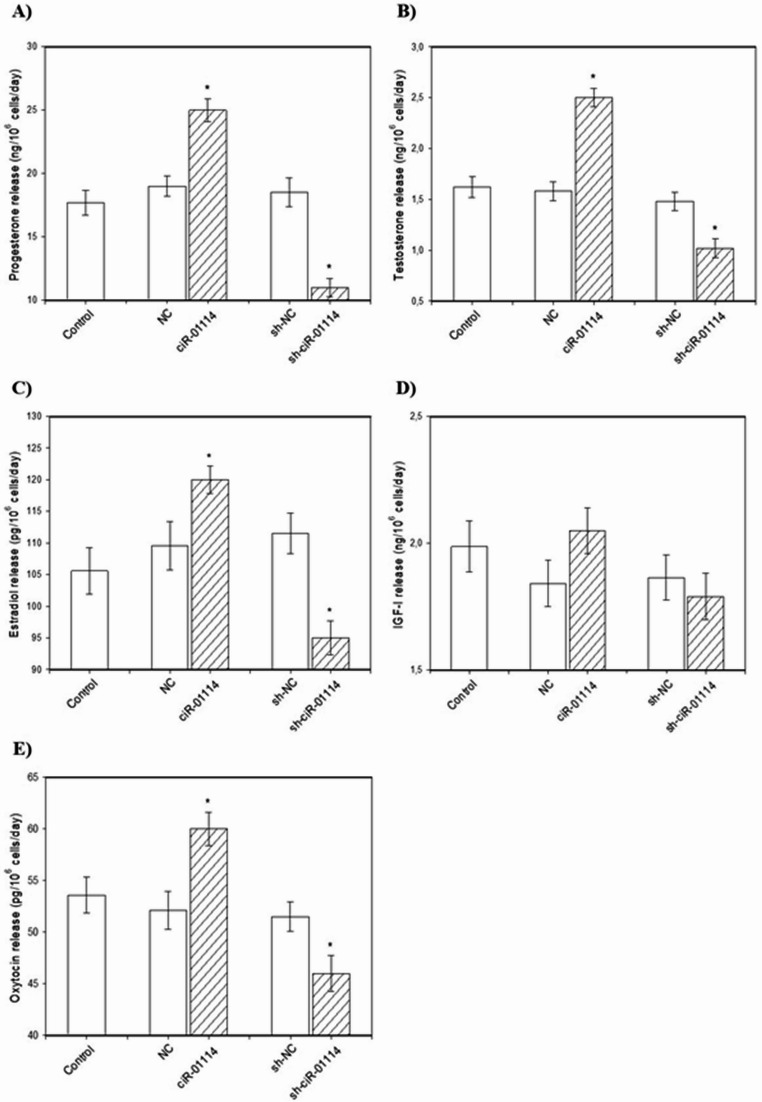
Effects of ciR-01114 overexpression and ciR-01114 suppression on the release of progesterone [**A**], testosterone [**B**], estradiol [**C**], IGF-I [**D**], and oxytocin [**E**] in cultured porcine ovarian granulosa cells. The results show (*) the effects of ciR-01114 overexpression or ciR-01114 suppression, with a significant (*P* < 0.05) difference between cells transfected with an empty pEX-3 vector as a negative control (NC) or the shRNA negative control (sh-NC) and cells transfected with the ciR-01114 overexpression vector or ciR-01114 shRNA vector. The results are expressed as means ± SEM from at least 3 independent experiments

CiR-01114 overexpression augmented progesterone, testosterone, estradiol, and oxytocin release (Figs. 5A, B, C, E) but did not affect IGF-I output (Fig. 5D). Furthermore, sh-ciR-01114 had a suppressive effect on progesterone, testosterone, estradiol, and oxytocin release (Figs. 5A, B, C, E) but did not affect IGF-I secretion (Fig. 5D).

## Discussion

The cultured ovarian cells displayed signs of being alive and suitable for the study as indicated by the presence of molecules associated with cell growth and cell death, their ability to exclude Trypan blue, and their secretion of steroid and peptide hormones. Additionally, fluorescence microscopy and RT‒qPCR analyses were employed to confirm the efficiency and reliability of the ciR-01114 transfection method, as described previously [32].

### The role of ciR-01114 in the control of ovarian cell proliferation

Recent studies have highlighted the mammalian ovary as a rich source of thousands of circRNAs, including ciR-01114 [8]. However, its role in the control of ovarian functions remains unknown. Our research is the first to demonstrate that ciR-01114 is not only produced in the ovary but also plays a crucial role as a regulator of essential ovarian cell functions. Our experimental findings show that the transfection of porcine granulosa cells with the ciR-01114 overexpression vector resulted in a notable increase in the accumulation of proliferation-related proteins, such as PCNA and cyclin B1. Conversely, transfection with the shRNA knockdown vector for ciR-01114 (sh-ciR-01114) inhibited cell proliferation. Proliferation is a vital process in ovarian physiology and significantly contributes to folliculogenesis and oocyte growth [33]. The orchestrated modifications in the morphological and physiological attributes of granulosa cells during follicle formation are intricately associated with cellular proliferation. These in vitro results suggest a potential pro-proliferative effect of ciR-01114 on ovarian cells. CiR-01114 influences the proportion of PCNA (an endogenous promoter and marker of the S-phase of mitosis) [34] and cyclin B1 (which governs the transition from the G2 to M phase of the cell cycle) [35], suggesting that ciR-01114 exerts a stimulatory effect on mitosis at the G2 and S phases by modulating these proteins. Further research is needed to confirm these findings and to explore the precise molecular mechanisms by which ciR-01114 influences PCNA and cyclin B1 levels. While these in vitro findings suggest a potential role for ciR-01114 in promoting proliferation-related reproductive events such as ovarian folliculogenesis, oogenesis, follicular selection, and ovulation, further studies, including in vivo models, are necessary to validate these observations.

### The roles of ciR-01114 in the control of ovarian cell apoptosis

The relationship between cell proliferation and apoptosis in the ovary is complex, as both processes contribute to folliculogenesis and oocyte maturation [36]. Our study indicates that ciR-01114 may play an antiapoptotic role by inhibiting apoptosis in ovarian cells. Specifically, ciR-01114 overexpression resulted in decreased levels of pro-apoptotic proteins bax and caspase-3, suggesting a reduction in both cytoplasmic and mitochondrial apoptosis [37]. Additionally, the reduction of histone-associated DNA fragmentation further supports the role of ciR-01114 in preventing nuclear apoptosis in granulosa cells [38]. Conversely, knockdown of ciR-01114 with shRNA increased apoptosis-related proteins, confirming the antiapoptotic effect of ciR-01114. These findings highlight the potential dual role of ciR-01114 in balancing cell proliferation and apoptosis, processes that are critical for ovarian homeostasis. However, the precise molecular mechanisms underlying these effects remain unclear and warrant further investigation. For example, it is possible that ciR-01114 influences key signaling pathways involved in apoptosis, such as those mediated by caspases or mitochondrial dynamics. Further mechanistic studies are required to uncover how ciR-01114 modulates these apoptotic pathways.

### The role of ciR-01114 in controlling ovarian cell viability

CiR-01114 was found to enhance cell viability, likely due to its effects on both proliferation and apoptosis. Increased cell viability, observed with ciR-01114 overexpression, may result from an improved proliferation/apoptosis balance, with increased proliferation and decreased apoptosis. This finding suggests that ciR-01114 may help reduce follicular atresia, a process that leads to the regression of ovarian follicles, by promoting the survival of granulosa cells. Furthermore, ciR-01114 may support folliculogenesis and oogenesis, possibly leading to increased fertility [39]. CiR-01114 may influence ovarian cell viability through the modulation of the release of steroids and peptide hormones, which are key regulators of cellular viability, proliferation, and apoptosis, and the processes governing ovarian follicular development or atresia [24]. However, while these results are promising, the exact pathways by which ciR-01114 regulates ovarian cell viability, including its potential effects on signaling molecules and hormones, require further elucidation.

### The roles of ciR-01114 in controlling ovarian secretory activity

These in vitro findings suggest that ciR-01114 may enhance the release of progesterone, testosterone, estradiol, and oxytocin in porcine ovarian granulosa cells but does not appear to impact the output of IGF-I. Conversely, blocking ciR-01114 appears to lead to a decrease in these ovarian hormones. This study provides the first in vitro evidence of a potential role for ciR-01114 in modulating the secretory activity of porcine ovarian cells, specifically through its impact on steroid hormone and oxytocin release. The ability of ciR-01114 to modulate the release of these hormones suggests its potential involvement in regulating hormone-dependent ovarian processes. For example, progesterone serves as both a marker and a promoter of ovarian cell luteinization, whereas estradiol and oxytocin are critical for follicle recruitment, growth, and development. Additionally, ciR-01114 appears to influence testosterone release, which can be involved in ovarian follicular atresia [24]. These in vitro findings suggest a complex role for ciR-01114 in potentially modulating not only ovarian folliculogenesis but also ovarian follicular atresia and follicular cell turnover. Therefore, our data suggests that ciR-01114 may function as a modulator of ovarian follicle survival, growth, and luteinization. While these observations are intriguing, further research is required to validate these findings and to explore the precise mechanisms involved. The impact of ciR-01114 on these hormones may involve primary effects mediated through alterations in hormone precursors, with secondary effects potentially arising from these changes. For instance, progesterone, a precursor to testosterone, undergoes aromatization to produce estradiol [40]. Thus, alterations in testosterone and estradiol levels might be attributable to modifications in their precursor production. Additionally, testosterone and estradiol can implement negative feedback mechanisms to regulate and limit excessive progesterone production. Furthermore, ciR-01114 may play a role in modulating ovarian steroidogenesis through oxytocin, a significant regulator of steroid hormone synthesis [24]. This could influence the release of progesterone, testosterone, and estradiol by modulating oxytocin production, and vice versa. On the other hand, the apparent lack of influence of ciR-01114 on the release of IGF-I, another important stimulator of ovarian steroidogenesis [24], suggests that the observed changes in steroid hormone levels are likely not mediated by IGF-I. Further studies are needed to dissect these complex regulatory networks and to elucidate the broader physiological and biochemical effects of ciR-01114 on ovarian function and hormone regulation. Specifically, future research should focus on identifying the specific signaling pathways and molecular targets through which ciR-01114 exerts its effects on steroidogenesis. This will involve techniques such as gene expression analysis, proteomics, and the use of specific inhibitors or activators of signaling pathways. Additionally, in vivo studies will be crucial to confirm the relevance of these in vitro findings and to assess the potential of ciR-01114 as a therapeutic target.

## Conclusion, limitations of the present study, and possible directions for future studies

Our findings here reveal for the first time that ciR-01114 functions as a potent physiological promoter of porcine ovarian cell viability, proliferation, and the release of steroid hormones and oxytocin while concurrently acting as an inhibitor of apoptosis. Furthermore, our research suggests that ciR-01114 is not only produced in the ovaries of pigs [23] but also may promote basic ovarian cell functions, potentially regulating ovarian follicle survival, growth, and follicular luteinization.

From a practical perspective, these results suggest that ciR-01114 regulators could be beneficial for promoting ovarian follicular growth and development as well as synchronizing the cell cycle. For example, the application of ciR-01114 for farm animal reproductive processes and sh-ciR-01114 for temporarily blocking internal cycles for their synchronization might be hypothesized. Moreover, pigs may serve as valuable models for identifying novel regulators of reproductive processes in other species, including humans. The potential application of ciR-01114 in inducing ovulation and treating infertility in assisted reproduction cannot be excluded. Moreover, ciR-01114 has potential as a diagnostic and predictive marker for certain disorders and as a basis for developing novel therapies for hormone-related syndromes and tumorigenesis where suppression of ovarian cell functions is needed. However, it is important to acknowledge that these conclusions are preliminary and based on in vitro models. To validate and further elucidate these findings, additional research incorporating both in vitro and in vivo animal models, as well as clinical studies, is essential.

Future research directions should include: (1) Investigating the mechanisms underlying the stimulatory effects of ciR-01114 on testosterone release and its role in IGF-I-independent steroid hormone regulation; (2) exploring the signaling pathways and gene targets of ciR-01114, focusing on their physiological functions and potential applications in reproductive science; (3) determining the specific expression patterns of ciR-01114 under various ovarian physiological and pathological conditions, which could serve as diagnostic markers for evaluating these disorders; (4) developing reliable quantitative methods for measuring this circRNA to characterize and predict the state of specific tissues and cells; and (5) exploring novel therapeutic strategies for ovarian disorders by integrating circRNA overexpression or suppression vectors with traditional pharmaceutical treatments. It is possible that ciR-01114 could be effective in treating reproductive disorders in women if further experiments confirm similar effects. Such research could enhance our understanding of the molecular mechanisms controlling female reproductive processes and the broader implications of ciR-01114 for reproductive health and medicine.

## Data Availability

The authors affirm that the data backing up the conclusions of this study can be found in the article.
